# Notes and Notices

**Published:** 1890-04

**Authors:** 


### NOTES AND NOTICES.

Corrections.—March number, page 119, sixth line from the bottom, for organization read *organism*. Same page, second line from bottom, for 51 M read 55 M. Page 120, thirteenth line from top, for 76 M read 70 M. Page 120, sixteenth line, for 51 M read 55 M.

Dr. William S. Gee, of Chicago, announces that he has associated with him in his practice Dr. H. C. Allen, of Ann Arbor, the well-known editor of *The Medical Advance*. Their office will be at at 5401 Jefferson Avenue, from which address *The Advance* will hereafter be issued.

Removals.—Dr. F. W. Grundman has removed from S. E. corner Jefferson Avenue and Washington Street, to 2344 Washington Street, St. Louis, Mo. Dr. J. Adams, from 150 Bays Street to 12 St. Patrick Street, Toronto, Canada. Dr. R. O. Harris, from Soldier, Kan., to Boonville, Mo. Dr. W. L. Reed, from 2309 to 2812 Washington Avenue, St. Louis. Dr. A. McNeil, from 220 Turk Street to 784 Van Ness Avenue, San Francisco. Dr. Clarence N. Payne, from Port Jervis, N. Y., to Bridgeport, Conn. Dr. H. C. Allen, from Ann Arbor, Michigan, to Hyde Park, Chicago. Dr. L. L. Helt, from Columbus, Ohio, to Franklin, Ohio.

Cholera has appeared at Bagdad, in Mesopotamia. Jews, Mohammedans, and Christians are fleeing before its grim features (under allopathic treatment) like chaff before the wind. If we have a visitation we predict that fewer lives will be lost under our treatment than under any other—for we have one of nature's laws to guide and strengthen us.

The American Institute of Homœopathy will hold its next annual meeting at Waukesha, Wisconsin, at the "Fountain-Spring House," on Monday, the 16th of June, at 7.30 p. m. The President, Alfred I. Sawyer, M. D., has issued a circular announcing the order of business, and a list of interesting papers to be read.

The Grace Hospital, Detroit, Mich.—The next regular competitive examination for the position of Junior Assistant to the House Surgeon of the Grace Hospital, will be held at the Hospital on Thursday, April 10th, 1890, at four o'clock p. m. Term, eighteen months: first six months as junior assistant and apothecary; second six months as senior assistant and ambulance surgeon; third six months as house surgeon. Applicant must show evidence of graduation from a recognized homœopathic college. All applications must be addressed to the President of the Medical Board, Grace Hospital, Detroit, Mich., and must be accompanied by a certificate of good moral character. All applications must be presented not later than April 1st, 1890.

Robert H. Sillman, *Superintendent.*

Extract from a letter of W. M. Thackeray, 1849.—"I am going to kill Mrs. Pendennis presently, and have her ill in this number. Minnie says, O papa! do make her well again; she can have a regular doctor and be almost dead, and then will come a homœopathic physician who will make her well, you know."

To Biological Students.—From a desire to verify his own researches as to the causes of failing nutrition in aging organisms, the undersigned hereby offers three cash prizes of $175, $125, and $100 for the best three comparative demonstrations, by means of microscopical slides, of the blood capillaries in young and in aged tissues, canine or human.

For particulars, address C. A. Stephens' Laboratory, Norway Lake, Maine.

THE DOCTOR IMPOSED ON.—Professor Bilroth, the famous Viennese surgeon, some time ago received a letter from a certain Jew in a small Russian town to come immediately and perform an operation. The professor in his answer stipulated for five thousand marks, which was promised him. The professor then repaired to the Russian town, and upon his arrival he was received by a number of Jews, who sorrowfully informed him that the gentleman that was to be operated upon had died, and had been buried already. And seeing that the professor felt perplexed and regretted the journey which he had made in vain, the Jews comforted him, saying: "There is yet some chance for you to make some money here. There are several sick men in our hospital who would require your services, for which each of them would willingly pay you one thousand marks."

The professor gladly accepted the offer, and after having performed about five operations the stipulated amount was handed to him. But a few minutes before starting for home the professor learned that he had resurrected the dead man. That worthy gentleman had been among the hospital patients cured for one thousand marks.—*Jewish Chronicle.*

NEW YORK STATE MEDICAL SOCIETY.—The State Medical Society in session at Albany, February 15th, unanimously adopted the following resolution:

"*Resolved*, That the members of the Medical Society of the State of New York, which has represented the medical profession of the State eighty-five years, without any reference to political opinions and affiliations, and merely guided by their scientific convictions as experts in medical matters and their sense of duty and the dictates of conscience as citizens, entreat and urge the members of the Legislature, without distinction of party or of local interest, to pass the bill referring to the pauper insane as presented by the State Charities Aid Association, and thus terminate a condition, the horrors of which, as detailed in the annual report of the State Commission in Lunacy, appear to be irretrievably connected with the county care of the insane."

The Society favor the passage of Senator Stadler's State Medical Board of Examiners' bill and recommend that the medical schools throughout the State add the study of Homœopathy to their respective courses.

About one hundred and seventy-five members sat down to the annual banquet of the Society at the Delavan House.

### FUN FOR DOCTORS.

Galligan—"Doctor, haven't you been attending an old man Gilfullaw?"
Doctor—"Yes."
"How is he to-day?"
"He is beyond the reach of medical assistance, I fear."
"What? Is he dying?"
"Oh! no; he's broke."—*Medical Times.*

Doctor—"Well, my fine little fellow, you have got quite well again? I was sure that the pills I left for you would cure you. How did you take them, in water or in cake?"
"Oh! I used them in my blow-gun."—*Fliegende Blatter.*

A sure remedy.—"Did you ever call upon Dr. Banquet professionally?"
"Yes, once. I was drowning."
"Drowning?"
"Yes. He diagnosticated my case on the instant, and wrote a prescription on a chip, which he threw into the water where I could get it."
"What was the prescription?"
"Swim."—*Life.*

